# Influence of protein concentration and quality in a canned diet on urine composition, apparent nutrient digestibility and energy supply in adult cats

**DOI:** 10.1186/s12917-018-1517-x

**Published:** 2018-07-25

**Authors:** Nadine Paßlack, Barbara Kohn, Marcus G. Doherr, Jürgen Zentek

**Affiliations:** 1 Institute of Animal Nutrition, Department of Veterinary Medicine, Freie Universität Berlin, Königin-Luise-Str. 49, 14195 Berlin, Germany; 2 Clinic of Small Animals, Department of Veterinary Medicine, Freie Universität Berlin, Oertzenweg 19b, 14163 Berlin, Germany; 3 Institute of Veterinary Epidemiology and Biostatistics, Department of Veterinary Medicine, Freie Universität Berlin, Königsweg 67, 14163 Berlin, Germany

**Keywords:** Cats, Dietary protein concentration, Dietary protein quality, Urine, Apparent digestibility

## Abstract

**Background:**

Protein concentration and quality in cat food can vary considerably, and the impact on feline urine composition and nutrient supply is of high practical relevance. In the present study, 6 canned diets with varying protein concentrations and qualities were fed to 10 healthy adult cats. Protein quality in the diet differed depending on the amount of collagen-rich ingredients. Hydroxyproline concentrations were 2.56–4.45 g/kg dry matter in the high quality and 3.76–9.44 g/kg dry matter in the low quality diets. Protein levels were 36.2, 43.3 and 54.9% in the high quality and 36.7, 45.0 and 56.1% in the low quality groups. Each diet was fed for 6 weeks, using a randomized cross-over design. In the last 2 weeks of each feeding period, urine and faeces of the cats were collected.

**Results:**

Renal calcium (Ca), oxalate (Ox) and citrate excretion were unaffected by the dietary protein concentration, possibly mediated by a high urine volume (24.2–34.2 ml/kg bodyweight (BW)/day) in all groups. However, renal Ox excretion was lower when the high quality diets were fed (*P* = 0.013). Urinary relative supersaturation (RSS) with calcium oxalate (CaOx) was low in general, but reduced in the high quality groups (*P* = 0.031). Urinary RSS values for magnesium ammonium phosphate (MAP) were high (2.64–5.00) among all groups. Apparent digestibility of crude protein and most minerals was unaffected by the different diets. Feed intake was higher in the low quality groups (*P* = 0.026), but BW of the cats did not differ depending on dietary protein quality. BW of the cats increased with increasing dietary protein concentrations (*P* = 0.003).

**Conclusion:**

In conclusion, a high protein canned diet might not be a specific risk factor for CaOx urolith formation in cats. In contrast, all diets resulted in high RSS MAP values, which might be critical concerning MAP crystallization. Protein quality had a minor, but significant impact on urine composition, necessitating further research on this subject. A lower energy supply when feeding a low protein quality can be assumed. Changes in BW were only small and require a careful interpretation.

**Electronic supplementary material:**

The online version of this article (10.1186/s12917-018-1517-x) contains supplementary material, which is available to authorized users.

## Background

Previous studies have raised the question whether high dietary protein concentrations or a low dietary protein quality might enhance the risk for calcium oxalate (CaOx) urolith formation in cats. In human subjects, a high intake of animal protein is associated with an increased renal calcium (Ca) and oxalate (Ox) excretion and a decreased renal citrate excretion [32]. On the contrary, high dietary protein concentrations were associated with beneficial effects for the prevention of urinary stones in cats, since an increased urine volume [9], a lower renal Ox excretion [34] or reduced urinary Ox concentrations [3] were observed. On the other hand, our previous study in cats [26] demonstrated that a high protein intake resulted in an increase in renal Ca and Ox excretion and urinary relative supersaturation (RSS) with CaOx as well as in decreased urinary citrate concentrations when compared to feeding diets with lower protein concentrations. Moreover, feeding a diet with a high protein level, but low protein quality decreased the renal Ca excretion in cats when data were compared with a high protein level-high protein quality group, while the renal Ox excretion was similar between these two groups [26]. This observation contrasts with findings from Dijcker et al. [4], where the renal Ox excretion was increased when synthetic hydroxproline, an amino acid found in higher amounts in collagen-rich dietary ingredients, was added to a diet for cats. Zentek and Schulz [34] also found a higher renal Ox excretion when cats were fed diets based on collagen tissue when compared to diets based on soy protein isolate or horsemeat.

To sum up these contradicting results of studies in cats, effects of dietary protein concentration and quality on urine composition seem to depend on additional dietary factors. It can be assumed that the protein source used for experimental diets might be one factor to consider. Moreover, type of diet, particularly the use of either a dry extruded or a canned diet, might affect urine output, as moisture intake by feed might modulate urine volume and concentration. Results obtained when feeding a dry food might therefore not be transferable for the use of canned diets. As previous studies used experimental diets with low moisture concentrations [4, 26], it was the aim of the present study to investigate the effects of varying protein concentrations and qualities in a canned diet on the urine composition of cats and to detect possible differences to the use of a dry food.

Besides the impact on the urine composition, the effect of a varying protein concentration and quality in a diet for cats on the apparent nutrient digestibility and energy supply requires further evaluation. Protein concentration and quality in diets for cats can vary considerably, and differences in nutrient digestibility can be assumed. Protein, but also mineral digestibility might be particularly interesting, since it has been demonstrated that the Ca and phosphorus (P) retention was reduced in growing kittens by high dietary protein concentrations [10]. Moreover, the calculation of metabolisable energy (ME) in cat food does not explicitly consider the quality of its ingredients [22]. It can therefore be hypothesised that variations in nutrient and energy supply occur when different dietary protein qualities are fed to cats. The present study aimed at investigating these aspects in more detail.

## Methods

### Study design

For the present study, cats and housing facilities of the Institute of Animal Nutrition, Freie Universität Berlin, were used. The animal care and use protocol was approved by the Animal Welfare Committee (Landesamt für Gesundheit und Soziales, Berlin, Germany, G 0138/12).

Six experimental diets were offered to 10 healthy adult cats (European shorthair; 82 ± 17 months old), using a randomized cross-over design. The diets differed in protein concentration and quality (Table 1). The diets with a high protein quality contained higher amounts of meat and blood meal when compared to the low protein quality diets. A lower protein quality was achieved by higher amounts of collagen-rich ingredients in the diets (trachea and greaves meal). At a comparable protein level, the hydroxyproline concentrations were always lower in the high protein quality diets than in the low protein quality diets (for high protein quality vs. low protein quality: 2.56 g/kg dry matter (DM) vs. 3.76 g/kg DM [low protein level]; 3.76 g/kg DM vs. 8.45 g/kg DM [medium protein level]; 4.45 g/kg DM vs. 9.44 g/kg DM [high protein level]). The hydroxyproline concentrations were used as a marker for protein quality of the experimental diets, as it has been demonstrated that hydroxyproline can be considered as a marker for dietary collagen content [1], which is a quality index of meat material [20].

**Table 1:** Nutrient analysis of the experimental diets^a,b^

		Low protein quality			High protein quality		
Analysed composition		36.7%	45.0%	56.1%	36.2%	43.3%	54.9%
Dry matter	g/kg	200	189	204	216	203	193
Crude protein	g/kg DM	367	450	561	362	433	549
Crude fat	g/kg DM	284	296	271	294	318	262
Crude fibre^c^	g/kg DM	3.14	7.05	10.5	6.75	4.46	6.78
Crude ash	g/kg DM	77.2	73.8	83.4	68.3	68.9	75.2
Ca	g/kg DM	11.2	11.4	12.6	10.7	11.2	11.8
P	g/kg DM	8.79	8.34	8.88	7.73	8.23	8.71
Na	g/kg DM	5.83	5.75	8.20	4.88	5.78	6.74
K	g/kg DM	11.1	9.35	12.4	8.75	8.58	8.67
Mg	g/kg DM	0.48	0.48	0.47	0.45	0.47	0.49
Ox	g/kg DM	0.25	0.28	0.32	0.32	0.34	0.35
Metabolisable energy^d^	MJ/kg DM	20.4	20.8	20.4	20.6	21.3	20.4
Nitrogen-free extracts	g/kg DM	269	173	74.7	269	176	107

^a^Ingredients in descending order (high protein quality diets): liver, meat, wheat flour, sunflower oil, blood meal, greaves meal, minerals, gelling and thickening agents, vitamins

^b^Ingredients in descending order (low protein quality diets): liver, trachea, wheat flour, meat, sunflower oil, greaves meal, blood meal, minerals, gelling and thickening agents, vitamins

^c^Additional analysis of total dietary fiber (TDF) revealed that the values were below the detection limit for all experimental diets. The analyses were performed according to the instructions of the manufacturer (Total Dietary Fiber Assay Kit, Megazyme u.c., Ireland)

^d^Calculated according to NRC [22]

Each diet was fed for 6 weeks. The daily amount of feed was calculated according to the energy recommendations for adult cats [22] in order to maintain body weight (BW). For each diet, a 31-day adaptation period was considered. During this period, cats were fed individually, but were housed in groups. Each adaptation period was followed by a 2 × 4-day sampling period with a 3-day rest period between. During the sampling period, cats were fed and housed individually in metabolic cages in order to collect urine and faeces. Cats were housed in groups, but fed individually in the rest period. At the end of each sampling period, blood was collected when cats were fasting.

The metabolic cages were prepared with cat litter boxes with plastic pellets as litter. The litter boxes were connected with urine collection containers. The urine could flow into these containers along a gradient of the litter boxes, while the faeces of the cats remained in the boxes. In order to inhibit bacterial growth in the urine, the collection containers were prepared with 3 drops chlorhexidine digluconate. Urine and faeces of the cats were collected twice a day (6.30 h and 12.30 h, prior feeding) and stored at − 20 °C for later analysis.

### Nutrient analysis

Crude nutrient, mineral and amino acid concentrations of the experimental diets are presented in Tables 1 and 2 (for the nutrient concentrations based on the dietary energy density, see Additional file 1: Table S1). Crude nutrient concentrations were analysed according to the directions of the Weende analysis of feed [23]. Crude fat analysis was modified as specified elsewhere [27]. Mineral concentrations in the diets were measured as described below for the faecal samples. Dietary amino acid concentrations were determined as follows: At first, diets were ground to a particle size of 0.5 mm. In case of methionine and cysteine determination, samples were oxidised prior to hydrolysis. The oxidation solution contained 0.5 ml H_2_O_2_ (30%), 4.5 ml formic acid (85%) and 25 μl phenol. The solution was mixed and placed for 1 h into a water bath (30 °C). Afterwards, the solution was placed into an ice bath for 5 min and was immediately used for the following oxidation of the samples. Therefore, 500 mg of the grounded sample was mixed with 5 ml of the oxidation solution. The mixture was placed into an ice bath in a refrigerator for 24 h. Afterwards, oxidation was stopped by adding 0.9 g sodium metabisulphite to the sample. As a next step, samples (oxidised samples in case of methionine and cysteine measurements; grounded samples for the measurement of other amino acids) were hydrolysed using hydrochloric acid (6 M). 500 mg of the samples were mixed with 25 ml hydrochloric acid (6 M) in a glass bottle and placed into a compartment dryer for 1 h at 110 °C. Afterwards, the glass bottles were sealed and placed for 23 h into the compartment dryer. The samples were air-dried and placed into an ice bath. Subsequently, 20 ml sodium hydroxide solution (7.5 mol/l) was added carefully to each sample in 5 steps (2 ml, 3 ml, 5 ml, 5 ml, 5 ml). A pH of 2.20 was achieved by adding either hydrochloric acid or sodium hydroxide to the samples. The samples were transferred into 100 ml graduated flasks and filled up with a sodium loading buffer, pH 2.20 (Biochrom Ltd., Cambridge, UK). This solution was mixed and 1.5 ml were transferred via a membrane filter into a vial. Samples were subsequently analysed using ion chromatography (Biochrom 20 Plus, Amersham Pharmacia Biotech, Piscataway, USA). A lithium column (High Performance) was used (Biochrom Ltd).

**Table 2:** Amino acid and taurine concentrations in the experimental diets

		Low protein quality			High protein quality		
		36.7%	45.0%	56.1%	36.2%	43.3%	54.9%
Aspartic acid	g/kg DM	26.5	33.3	38.7	25.1	33.8	42.9
Threonine	g/kg DM	14.2	17.8	20.2	14.1	18.3	23.6
Serine	g/kg DM	14.6	18.9	21.9	14.8	18.5	23.2
Glutamic acid	g/kg DM	35.9	40.0	49.0	35.7	38.9	44.1
Glycine	g/kg DM	36.0	39.1	52.6	28.0	23.8	36.6
Alanine	g/kg DM	18.9	26.6	32.9	17.6	23.1	30.3
Valine	g/kg DM	18.0	22.4	25.9	12.2	22.4	27.0
Isoleucine	g/kg DM	12.8	16.1	17.8	9.2	17.2	21.2
Leucine	g/kg DM	24.7	31.4	37.6	24.4	32.4	39.3
Tyrosine	g/kg DM	10.4	13.8	16.1	10.6	14.9	18.7
Phenylalanine	g/kg DM	13.9	18.3	21.6	14.1	18.8	24.1
Histidine	g/kg DM	12.0	15.4	15.5	10.9	17.1	20.8
Lysine	g/kg DM	21.6	28.3	33.6	20.9	29.6	36.5
Arginine	g/kg DM	17.3	24.9	30.5	15.4	23.6	31.2
Proline	g/kg DM	16.1	25.0	30.3	15.9	17.3	23.4
Methionine	g/kg DM	6.29	8.77	10.1	5.74	9.86	11.6
Cysteine	g/kg DM	6.47	7.67	8.93	6.63	8.75	10.4
Hydroxyproline	g/kg DM	3.76	8.45	9.44	2.56	3.76	4.45
Taurine	g/kg DM	5.32	4.36	3.90	4.13	3.81	3.85

Ox concentrations in the experimental diets were analysed as described below for the faecal samples.

### Urine analysis

The urinary pH was measured immediately after urine collection at 6.30 h and 12.30 h, using the Seven Multi pH meter (Mettler-Toledo GmbH, Schwerzenbach, Switzerland). Except for sodium (Na), magnesium (Mg) and potassium (K), urinary anion and cation concentrations were measured with an ion exchange HPLC system (Dionex DX-500 and Dionex DX-120; Dionex Corp., Sunnyvale, CA, USA), as described previously [25]. The data were analysed using Chromeleon Client, version 6.80 SP2 (Dionex Corp.). Urinary Na, Mg and K concentrations were measured using atomic absorption spectrometry (ContrAA 700; Analytik Jena AG, Jena, Germany). The urinary RSS values for CaOx and magnesium ammonium phosphate (MAP) were calculated with the Supersat Program [31].

### Faeces analysis

Faecal Ca, K, Na and Mg concentrations were determined using the flame atomic absorption spectrometer type vario 6 with an autosampler AS 52 (Analytik Jena AG, Jena, Germany). Details on sample preparation and analysis can be found elsewhere [25]. The P concentrations in the faeces of the cats were measured with a spectrophotometer (Ultrospec 2000, Pharmacia Biotech, Cambridge, UK), as described by Gericke and Kurmies [7]. The faecal Ox concentrations were measured by HPLC (Dionex DX-500; Dionex Corp., Sunnyvale, CA, USA), which included a gradient pump (GP50), an electrochemical detector (ED40), an autosampler (ICS-3000 AS; all Dionex Corp., Sunnyvale, CA, USA) and a column cooler (IGLOO-CIL; Esslab, Hadleigh, Essex, United Kingdom). A Dionex IonPac AS11-HC was used as analytical column, and a Dionex IonPac AG11-HC as a pre-column. The ion suppressor was a Dionex SRS ULTRA II 4-mm (all Dionex Corp.). Twenty ml hydrochloric acid (c = 1 mol/l) were mixed with 1 g of the dried faecal sample and incubated for 30 min in a water bath at 100 °C. Subsequently, 1 ml trichloroacetic acid (w = 0.3%) was added to the samples. A pH of 2.9–3.1 was achieved by adding either hydrochloric acid or sodium hydroxide. The solution was transferred into a 25 ml graduated flask and filled up with hydrochloric acid (pH 3.0). For the filtration of the supernatant, solid phase extraction columns (StrataX 33 μ Polymeric Reversed Phase, Phenomenex, Aschaffenburg, Germany) were used. The condition of the columns was carried out with 1 ml methanol, and the equilibration with 1 ml hydrochloric acid (pH 3.0). Subsequently, 1 ml sample was added to the column, followed by 1 ml hydrochloric acid (pH 3.0).

### Blood analysis

Urea and creatinine concentrations in the serum of the cats were measured using an automatic method (Konelab 60 i Thermo Fisher Scientific, Thermo Electron GmbH, Dreieich, Germany).

### Statistical analysis

As the baseline information needed to generate meaningful sample size estimates was not available for most of the parameters, a formal sample size calculation was not performed. The sample size, however, was based on previous studies in cats evaluating dietary effects on urinary and faecal parameters (e.g. [3, 4, 19, 25–28]).

Data were analysed using SPSS 22 (SPSS Inc., Chicago, Illinois, USA). A repeated-measures ANOVA was performed for each parameter, considering dietary protein concentration and protein quality as within-subject factors. For each dependent variable, a separate ANOVA model was constructed. An adjustment for assessing the dietary effects on the different parameters was not foreseen, as they were considered to be independent hypotheses to be statistically tested. In case of a significant interaction between dietary protein concentration and quality, linear and quadratic polynomial contrasts for protein concentration were separately calculated for the three high protein quality and the three low protein quality groups. If the interaction between protein concentration and protein quality was not significant, linear and quadratic polynomial contrasts for protein concentration and linear polynomial contrasts for protein quality were calculated for the averaged measurements (high and low protein quality groups were examined together at protein levels). The data are presented in Tables as means and standard error of means. The alpha level of statistical significance was set to *P* <  0.05.

## Results

### Animal health and performance

One cat had to be removed from the study due to health problems, which were not associated with feeding the experimental diets. This cat was replaced by another cat of the Freie Universität Berlin with a comparable BW.

The BW of the cats increased with increasing dietary protein concentrations, independently of the dietary protein quality (*P* = 0.003) (Table 3). The feed intake of the cats was higher when the low protein quality diets were fed (*P* = 0.026) (Table 4).

**Table 3:** Body weight (kg), urine volume (ml/kg body weight/day), urinary pH, urine composition (mg/l) and relative supersaturation (RSS) of the urine of cats fed diets with varying protein concentrations and qualities. *n* = 10 / diet, Mean and SEM

								*P* values (polynomial contrasts)							
	Low protein quality			High protein quality			SEM	Interaction	Low protein quality		High protein quality		Protein concentration		Protein quality
	36.7%	45.0%	56.1%	36.2%	43.3%	54.9%			Lin.	Quadr.	Lin.	Quadr.	Lin.	Quadr.	
Body weight	4.05	4.30	4.34	4.15	4.19	4.24	0.16	0.873	–	–	–	–	**0.003**	0.442	0.783
Urine volume	28.0	34.2	31.5	25.2	24.2	27.8	1.45	**0.037**	0.427	0.103	**0.010**	0.571	–	–	–
Urinary															
pH (6.30 h)	8.45	8.30	8.44	8.49	8.31	8.44	0.05	0.841	–	–	–	–	0.680	0.284	0.899
pH (12.30 h)	8.18	8.36	8.30	8.25	8.16	8.19	0.05	0.287	–	–	–	–	0.762	0.456	0.351
Ca	8.98	9.65	13.6	12.4	9.96	8.87	1.01	0.247	–	–	–	–	0.431	0.264	0.363
P	1055	1110	1070	931	1022	1027	42.7	0.604	–	–	–	–	0.103	0.698	0.302
Mg	11.3	6.06	5.54	7.11	6.03	5.87	0.62	0.483	–	–	–	–	**0.040**	0.304	0.430
K	3415	3546	3707	3176	3154	3528	97.8	0.393	–	–	–	–	0.175	0.913	0.352
Na	2041	2033	3122	2149	2267	2415	124	0.324	–	–	–	–	**0.006**	0.499	0.563
Urea	54.4	61.3	77.8	57.5	71.8	74.7	2.67	0.103	–	–	–	–	**< 0.001**	0.646	0.995
Creatinine	1239	1241	1491	1438	1561	1513	42.5	0.070	–	–	–	–	0.059	0.696	**0.014**
Sulphate	830	977	1301	989	1301	1607	54.1	0.820	–	–	–	–	**< 0.001**	0.495	**0.022**
Ox	59.7	54.8	56.1	54.2	51.1	45.3	1.61	**0.032**	0.524	0.563	**0.003**	0.808	–	–	–
Citrate	202	198	195	308	190	180	17.9	0.192	–	–	–	–	0.150	**0.022**	0.144
Ammonium	394	684	837	707	991	1138	98.0	0.845	–	–	–	–	**0.005**	0.663	0.058
RSS															
CaOx	0.88	0.82	1.16	0.92	0.79	0.59	0.09	0.198	–	–	–	–	0.882	0.475	**0.031**
MAP	3.29	2.64	3.44	4.41	4.70	5.00	0.90	0.772	–	–	–	–	0.515	0.942	0.322

**Table 4:** Feed intake (g/kg body weight/day) and renal excretion (mg/kg body weight/day) of cats fed diets with varying protein concentrations and qualities. *n* = 10 / diet, Mean and SEM

								*P* values (polynomial contrasts)							
	Low protein quality			High protein quality			SEM	Interaction	Low protein quality		High protein quality		Protein concentration		Protein quality
	36.7%	45.0%	56.1%	36.2%	43.3%	54.9%			Lin.	Quadr.	Lin.	Quadr.	Lin.	Quadr.	
Feed intake	55.3	58.1	54.2	48.1	50.4	56.7	1.53	0.152	–	–	–	–	0.110	0.953	**0.026**
Renal excretion															
Ca	0.25	0.36	0.39	0.34	0.25	0.24	0.04	0.463	–	–	–	–	0.265	0.404	0.141
P	29.2	39.3	29.6	21.8	23.7	28.5	1.76	0.308	–	–	–	–	**0.020**	0.437	0.088
Mg	0.32	0.19	0.15	0.18	0.13	0.17	0.02	0.265	–	–	–	–	0.098	0.251	0.239
K	96.5	118	122	75.4	75.1	99.2	6.53	0.052	–	–	–	–	**0.026**	0.540	**0.041**
Na	55.1	66.8	99.6	50.2	53.9	67.1	5.04	0.641	–	–	–	–	**0.003**	0.881	0.150
Ox	1.68	1.86	1.59	1.27	1.22	1.26	0.07	0.499	–	–	–	–	0.702	0.484	**0.013**
Citrate	5.40	7.48	6.20	7.53	4.48	4.83	0.64	0.629	–	–	–	–	0.333	0.186	0.922
Ammonium	10.8	23.0	23.7	18.0	21.7	30.3	2.26	0.224	–	–	–	–	**0.003**	0.683	0.077
Creatinine	33.6	38.6	40.8	32.9	36.3	41.6	0.90	0.164	–	–	–	–	**<0.001**	0.802	0.543
Urea	1.54	2.02	2.12	1.31	1.69	2.04	0.09	0.485	–	–	–	–	**<0.001**	0.582	**0.045**
Sulphate	23.0	32.5	35.7	24.1	30.9	44.7	1.61	**0.014**	**<0.001**	0.251	**<0.001**	**0.047**	–	–	–

### Urinary parameters

The urine volume of the cats was comparable when feeding the diets with the low protein quality, but increased with increasing dietary protein concentrations when the high protein quality diets were fed (*P* = 0.010). The urinary pH ranged between 8.16–8.49 and was not affected by the dietary protein concentration or quality (*P* > 0.05).

The urinary Ca concentrations and renal Ca excretion of the cats were neither affected by the protein level nor by the protein quality of the experimental diets (*P* > 0.05). The urinary Ox concentrations were comparable in the low protein quality groups, but decreased with increasing dietary protein concentrations in the high protein quality groups (*P* = 0.003). When the renal Ox excretion was calculated, values were generally lower in the high protein quality groups when compared to the low protein quality groups (*P* = 0.013). Moreover, urinary RSS CaOx values were lower when the diets with the high protein quality were fed (*P* = 0.031).

The urinary P concentrations were unaffected by the dietary protein concentration and quality (*P* > 0.05), but the renal P excretion was the lowest when the diets with the low protein levels were fed (*P* = 0.020). The Mg concentrations in the urine of the cats decreased with increasing dietary protein levels (*P* = 0.040), but the renal Mg excretion was unaffected by the experimental diets (*P* > 0.05). The ammonium concentrations in the urine (*P* = 0.005) and renal ammonium excretion of the cats (*P* = 0.003) increased with increasing dietary protein concentrations, independently of the protein quality of the diets. The urinary RSS MAP values were high and ranged between 2.64–5.00, however, no effect of the dietary protein concentration or quality was detected (*P* > 0.05).

The urea concentrations in the urine of the cats (*P* <  0.001) and renal urea excretion (*P* < 0.001) increased with increasing protein concentrations in the experimental diets. In addition, the renal urea excretion was generally higher when the diets with the low protein quality were fed (*P* = 0.045).

The urinary creatinine concentrations were higher in the high protein quality groups (*P* = 0.014). When the renal creatinine excretion was calculated, an increase was demonstrated with increasing protein concentrations in the diets, independently of the dietary protein quality (*P* < 0.001).

The sulphate concentrations in the urine increased by approximately 60% with increasing dietary protein concentrations (*P* < 0.001) and were generally higher when feeding the diets with the high protein quality (*P* = 0.022). The renal sulphate excretion of the cats increased in the low protein quality groups by approximately 55% when the dietary protein concentrations increased (*P* < 0.001). In the high protein quality groups, the renal sulphate excretion increased by nearly 86% with higher dietary protein levels (*P* < 0.001).

Only small variations in the urinary citrate concentrations were observed depending on the dietary protein levels (quadratic contrast: *P* = 0.022). The renal citrate excretion was unaffected by the protein concentration and quality of the experimental diets (*P* > 0.05).

The urinary K concentrations were unaffected by the experimental diets (*P* > 0.05). The renal K excretion increased with increasing dietary protein levels (*P* = 0.026) and was generally higher when the low protein quality diets were fed (*P* = 0.041).

The Na concentrations in the urine of the cats (*P* = 0.006) and renal Na excretion (*P* = 0.003) increased with increasing protein concentrations in the diets.

### Faecal parameters

The cats had a higher faecal volume when the protein concentration in the experimental diets increased (*P* < 0.05) (Table 5). The DM of the faeces was higher when the cats received the diets with the high protein quality (*P* = 0.012).

**Table 5:** Amount of faeces, dry matter (DM) of the faeces and crude protein (CP) and mineral concentrations in the faeces of cats fed diets with varying protein concentrations and qualities. *n* = 10 / diet, Mean and SEM

								*P* values (polynomial contrasts)							
	Low protein quality			High protein quality			SEM	Interaction	Low protein quality		High protein quality		Protein concentration		Protein quality
	36.7%	45.0%	56.1%	36.2%	43.3%	54.9%			Lin.	Quadr.	Lin.	Quadr.	Lin.	Quadr.	
Amount of faeces (g/kg body weight/day)	2.85	3.86	3.58	2.79	2.83	3.77	0.17	0.054	–	–	–	–	**0.026**	0.688	0.206
DM of the faeces (%)	33.8	33.5	35.1	40.9	37.5	36.0	0.98	0.796	–	–	–	–	0.339	0.984	**0.012**
Amount of faeces (g DM/kg body weight/day)	0.93	1.22	1.20	1.01	1.03	1.30	0.04	0.143	–	–	–	–	**0.023**	0.767	0.907
Faecal concentration (mg/g DM)															
CP	453	495	460	407	445	480	6.27	0.111	–	–	–	–	**0.001**	0.407	0.084
Ca	80.0	74.3	91.1	91.6	91.1	89.0	2.52	0.496	–	–	–	–	0.195	0.640	0.339
P	35.9	36.1	40.0	42.5	43.3	42.0	0.88	0.441	–	–	–	–	0.262	0.885	**0.013**
Mg	3.81	3.78	3.48	4.06	4.07	3.79	0.06	0.792	–	–	–	–	**0.042**	0.161	**0.010**
K	3.40	3.22	2.56	3.06	2.47	2.46	0.11	0.460	–	–	–	–	**0.020**	0.249	**0.049**
Na	3.30	3.28	3.20	2.26	2.62	2.87	0.14	0.978	–	–	–	–	0.160	0.944	**0.002**
Ox	0.84	0.78	0.79	0.96	0.83	0.75	0.02	0.141	–	–	–	–	**<0.001**	0.518	0.217

The faecal crude protein (CP) concentrations (*P* = 0.001) and faecal CP excretion (*P* = 0.004) was lowest when the diets with the low protein levels were fed and increased at higher dietary protein levels (Tables 5 and 6).

**Table 6:** Faecal excretion (mg/kg body weight/day) and apparent digestibility (App. digestib.) (%) of crude protein (CP) and minerals of cats fed diets with varying protein concentrations and qualities. *n* = 10 / diet, Mean and SEM

								*P* values (polynomial contrasts)							
	Low protein quality			High protein quality			SEM	Interaction	Low protein quality		High protein quality		Protein concentration		Protein quality
	36.7%	45.0%	56.1%	36.2%	43.3%	54.9%			Lin.	Quadr.	Lin.	Quadr.	Lin.	Quadr.	
Faecal CP excretion	423	600	553	418	458	625	20.9	0.076	–	–	–	–	**0.004**	0.524	0.609
App. digestib. of CP	89.7	87.7	90.7	89.0	89.7	89.5	0.30	0.133	–	–	–	–	0.376	0.117	0.943
Faecal Ca excretion	74.0	92.5	109	93.0	93.7	117	4.71	0.522	–	–	–	–	**0.012**	0.871	0.411
App. digestib. of Ca	40.8	22.0	18.0	17.3	19.2	8.85	3.32	0.394	–	–	–	–	0.086	0.547	0.257
Faecal P excretion	33.7	44.5	47.8	43.1	44.2	54.8	1.92	0.305	–	–	–	–	**0.018**	0.895	0.123
App. digestib. of P	65.6	49.2	49.1	46.8	47.5	41.6	1.95	0.165	–	–	–	–	0.062	0.274	**0.038**
Faecal Na excretion	3.12	4.09	3.70	2.43	2.74	3.81	0.21	0.260	–	–	–	–	0.009	0.683	**0.005**
App. digestib. of Na	95.4	93.7	95.8	95.5	95.5	94.8	0.24	0.057	–	–	–	–	0.963	0.122	0.153
Faecal Mg excretion	3.47	4.59	4.23	3.99	4.14	4.94	0.15	0.121	–	–	–	–	0.054	0.496	0.243
App. digestib. of Mg	34.9	10.3	14.0	13.1	13.6	6.01	2.74	0.237	–	–	–	–	0.123	0.280	0.119
Faecal K excretion	3.15	3.95	3.07	3.16	2.63	3.29	0.18	0.069	–	–	–	–	0.612	0.996	0.244
App. digestib. of K	97.5	96.2	97.7	96.6	97.1	96.6	0.13	**0.005**	0.314	**0.001**	0.698	**0.022**	–	–	–
Faecal Ox excretion	0.80	0.94	0.96	0.98	0.87	0.98	0.04	0.330	–	–	–	–	0.458	0.818	0.389

The Ca concentrations in the faeces of the cats were unaffected by the dietary protein concentration and quality (*P* > 0.05). However, when the faecal Ca excretion was calculated, increased values with increasing dietary protein concentrations were observed (*P* = 0.012), independently of the protein quality in the experimental diets.

The faecal P concentrations were generally higher when the high protein quality diets were fed (*P* = 0.013), while the faecal P excretion increased with increasing dietary protein concentrations (*P* = 0.018), both in the high and low protein quality groups.

The Ox concentrations in the faeces of the cats decreased with increasing protein levels in the experimental diets (*P* < 0.001), however, the faecal Ox excretion was not affected by the dietary protein concentration or quality (*P* > 0.05).

Both, the Mg and K concentrations in the faeces of the cats decreased with increasing dietary protein levels (*P* = 0.042 and *P* = 0.020, respectively). The values for Mg were higher and the values for K were lower when feeding the high protein quality diets (*P* = 0.010 and *P* = 0.049, respectively). The faecal Mg and K excretion were unaffected by the experimental diets (*P* > 0.05).

The faecal Na concentrations (*P* = 0.002) and faecal Na excretion (*P* = 0.005) of the cats were higher when the diets with the low protein quality were fed.

### Apparent nutrient digestibility

The apparent digestibility of minerals was only slightly affected by the experimental diets. The apparent digestibility of P was generally higher in the low protein quality groups (*P* = 0.038). Feeding the diet with a moderate protein level resulted in the lowest (*P* = 0.001) or highest (*P* = 0.022) apparent digestibility of K in the low protein quality or the high protein quality groups, respectively. The apparent digestibility of CP, Ca, Na and Mg was not affected by the dietary protein concentration and quality (*P* > 0.05).

### Blood parameters

The urea concentrations in the blood of the cats increased (*P* < 0.001) and the creatinine concentrations decreased (*P* = 0.004) with increasing dietary protein levels, independently of the dietary protein quality (Table 7). All values were within the reference range for cats (Clinic of Small Animals, Freie Universität Berlin).

**Table 7:** Urea and creatinine concentrations in the blood of cats fed diets with varying protein concentrations and qualities. *n* = 10 / diet, Mean and SEM

								*P* values (polynomial contrasts)							
	Low protein quality			High protein quality			SEM	Interaction	Low protein quality		High protein quality		Protein concentration		Protein quality
	36.7%	45.0%	56.1%	36.2%	43.3%	54.9%			Lin.	Quadr.	Lin.	Quadr.	Lin.	Quadr.	
Urea (mmol/l)	7.99	8.77	9.56	7.89	8.45	9.60	0.17	0.453	–	–	–	–	**< 0.001**	0.539	0.839
Creatinine (μmol/l)	151	153	142	160	150	149	2.69	0.432	–	–	–	–	**0.004**	0.711	0.112

Reference range for urea: 5.0–11.3 mmol/l; reference range for creatinine: 0–168 μmol/l (Clinic of Small Animals, Freie Universität Berlin)

## Discussion

The protein concentration and quality can vary considerably in diets for cats. In this context, however, only few studies have investigated diet-dependent effects on the urine composition, particularly with regard to urinary stone formation. In our previous study [26], a high protein diet was associated with specific risk factors for CaOx urolith formation in cats. We observed an increased renal Ca and Ox excretion, higher urinary Ca concentrations and RSS CaOx values and lower urinary citrate concentrations when a dry extruded diet with 57% CP was fed. These observations correspond with data in human subjects consuming high amounts of animal protein [32]. An increase of the renal Ca excretion and a decrease of the renal citrate excretion by a high protein intake is often explained by an “acid load”, resulting from an enhanced oxidation of sulphur-containing amino acids and an associated release of protons [33]. An acidification of the organism might reduce the Ca reabsorption and enhance the citrate reabsorption in the kidneys [32]. In the present study, however, no effect of the dietary protein concentration on the urinary Ca and citrate excretion could be observed. A possible explanation might be the high urine volume (24.2–34.2 ml/kg BW/day) of the cats and therefore a diluting effect of the canned diets. In our previous study [26], feeding dry extruded diets resulted in markedly lower urine volumes of the cats (9.86–17.0 ml/kg BW/day). Although the urinary sulphate concentrations increased with increasing dietary protein levels in the present study, indicating an enhanced oxidation of sulphur-containing amino acids, no “acid load” could be observed, as the urine pH was high (8.16–8.49). Urinary pH values < 6.25 are assumed to be associated with a higher risk for CaOx precipitation [14, 24]. It has been demonstrated that a high-protein diet (55% CP in DM) resulted in a lower urine pH (6.63) in cats when compared to a moderate protein diet (29% CP in DM), which led to a urine pH of 7.25 [5]. Moreover, a moderate protein diet (32.4% CP in DM) based on meat meal resulted in a higher urine pH (7.99) than a diet with a moderate protein level (30.9% CP in DM) based on maize gluten meal (7.08) [6]. Based on these studies, it can be concluded that both dietary protein concentration and quality can affect the urine pH in cats. The data of Funaba et al. [6] also indicate that animal protein does not necessarily result in an “acid load”, as described in human nutrition [32]. In our previous study [26], pH values of the feline urine were markedly lower (6.34–6.66) when diets with comparable protein concentrations as in the present study were fed. However, the dietary protein sources markedly differed between these studies. The observed higher urine volume in the present study might also have contributed to the higher urine pH of the cats, since urinary concentrations of pH affecting anions and cations were lower. Overall, the data on the renal Ca and citrate excretion and urine pH could not demonstrate a specific risk for the crystallization of CaOx uroliths by varying protein concentrations and qualities in a canned diet for cats. It should, however, be noted that the study was carried out in healthy research cats. The results might therefore be of particular relevance for healthy (pet) cats, whilst the clinical importance should be verified in diseased or predisposed cats in future investigations.

An increased renal Ox excretion by a high intake of animal protein might derive from an enhanced endogenous Ox synthesis from specific amino acids [32]. In a study by Dijcker et al. [4], the addition of synthetic hydroxyproline to a diet resulted in an increased urinary Ox excretion in cats. Zentek and Schulz [34] also found a higher renal Ox excretion when cats received diets based on collagen tissue (rich in hydroxyproline) when compared to diets based on horsemeat or soy protein isolate. However, feeding diets with different amounts of collagen tissue resulted in a higher urinary Ox excretion when the diet with the low amount of collagen tissue was fed, indicating that other dietary factors than specific amino acids might affect the endogenous Ox synthesis [34]. In our previous study in cats [26], the renal Ox excretion increased with increasing dietary protein concentrations (*P* < 0.05). Feeding high protein diets with varying amounts of collagen-rich greaves meal could, however, not affect the renal Ox excretion [26]. In the present study, the urinary Ox concentrations decreased with increasing dietary protein levels in the high protein quality groups, but were unaffected by the dietary protein level in the low protein quality groups. When the renal Ox excretion was calculated, lower values were detected when the high protein quality diets were fed, independently of the dietary protein concentrations. In this context, it should not go unmentioned that the dietary Ox concentrations were low in general, but slightly higher in the high protein quality diets than in the low protein quality diets. This might be attributed to necessary variations in the diet composition at different protein levels, but also reflects a limitation of the study. However, since the renal Ox excretion was lower in the high protein quality groups, this effect can possibly be attributed to differences in the endogenous Ox synthesis and not to the dietary Ox intake. Nevertheless, considering the described contradicting findings concerning the effects of the dietary protein level and quality on the urinary Ox excretion in cats, no clear relationship between these parameters has been identified. It might be possible that other dietary factors interfered with the urinary Ox excretion of the cats in the studies described, making further investigations necessary. In addition, it should be noted that the present study did not use a standard method for the determination of the dietary protein quality. As such, the protein efficiency ratio, biological value, net protein utilisation, and protein digestibility corrected amino acid score have been described [12]. In the present study, however, the hydroxyproline concentration in the diets has been used as a proxy for the amount of collagen tissue in the diets. Although this methodological approach might be a limitation of the study, it should be considered that hydroxyproline has been established as an index quality of meat material [20]. Thus, a differentiation between the high and low protein quality diets was ensured. Nevertheless, future studies carried out with further methods for determining dietary protein quality might clarify its effects on the urine composition of cats in more detail.

The calculation of the urinary RSS for CaOx demonstrated lower values when the cats received the diets with a higher dietary protein quality, independently of the protein level of the diet. This observation might be explained by the unaffected urinary Ca and Ox excretion depending on the dietary protein level, but the generally lower renal Ox excretion in the high protein quality groups. However, it should be considered that the RSS CaOx values were low, reaching values between 0.82–1.16 (low protein quality groups) and 0.59–0.92 (high protein quality groups). Values < 1 are considered to represent an undersaturated urine, where no new CaOx urolith formation can occur and existing CaOx uroliths cannot grow [13]. Values between 1 and 12 indicate a metastable stage, where no new CaOx urolith formation is possible, but already existing CaOx uroliths can grow [13]. RSS CaOx values > 12 are reached in the case of an oversaturated urine, where new CaOx urolith formation can occur and existing CaOx uroliths can grow [13]. Although generally lower RSS CaOx values were detected when the cats of the present study received the high protein quality diets, it should be considered that, except for the high protein level-low protein quality group, all values were < 1 and therefore represent an undersaturated urine. The RSS CaOx value in the high protein level-low protein quality group was only slightly higher (1.16). Overall, based on the measured RSS CaOx values, no specific risk for CaOx urolith formation can be assumed by the protein level or quality in a canned diet for cats. As described above, this might especially apply for healthy (pet) cats, while the generalisability for diseased or predisposed cats should be confirmed in future clinical investigations.

In contrast, urinary RSS MAP values found in the present study indicate that the cats were at risk to develop MAP uroliths, although no dependence on dietary protein concentration or quality could be detected. The RSS MAP values ranged between 2.64–3.44 (low protein quality groups) and 4.41–5.00 (high protein quality groups) and therefore represent an oversaturated urine [13]. For MAP, RSS values < 1 indicate that the urine is undersaturated and values between 1 and 2.5 represent a metastable stage [13]. The high values found in the present study might especially be explained by the high urinary ammonium concentrations, resulting from the dietary protein concentrations, independently of the protein quality of the diets. These results are of high practical relevance for healthy, but probably also for predisposed or diseased cats, demonstrating that an increased water intake by diets with a high moisture content cannot solely be considered as preventive factor for MAP urolith formation.

Although the present study could demonstrate that the dietary protein level did not affect the urinary Ca, Ox and citrate excretion in cats, other nutritional factors of the experimental diets should be critically discussed in the context of CaOx urolith formation. In particular, dietary mineral concentrations might be relevant. Studies in humans and rats have demonstrated that Ca and Mg can bind Ox in the intestine and therefore reduce its renal excretion [17, 18, 21, 29]. In the present study, however, the dietary Ca and Mg concentrations were comparable among the diets and no impact on the renal Ox excretion can therefore be assumed. Further studies have focussed on dietary Na and K, demonstrating an increased urine volume at high dietary Na intakes in cats [11, 28]. An increased urine volume and its diluting effect might be beneficial for urinary stone prevention [2]. Human studies have indicated that high dietary K levels can reduce the renal Ca excretion [15, 16], while this effect was not observed in cats [27]. In this study [27], however, increased urinary citrate concentrations were associated with an increased K intake. The present experimental diets showed some variations in the Na and K concentrations, possibly due to modifications of the dietary composition at different protein levels. However, the urine volume of the cats was high in general and not associated with variations in the dietary Na concentrations. Moreover, the renal Ca and citrate excretion of the cats remained unaffected by the experimental diets, independently of the dietary K concentration. Overall, it can be assumed that the unaffected renal Ca, Ox and citrate excretion by varying dietary protein levels was not influenced by additional nutritional factors of the experimental diets used in the present study.

Another aspect of this study was to investigate the impact of the dietary protein quality on the apparent nutrient digestibility and energy supply in cats. The calculation of ME in cat food does not explicitly consider the dietary protein quality [22]. However, variations in protein digestibility might be associated with differences in the energy intake. In the present study, a higher amount of collagen-rich dietary ingredients did not affect the apparent digestibility of CP. The calculated values were close to 90% in all groups. The apparent digestibility (%) of the nutrients was calculated as follows: (nutrient intake (mg/d) – faecal nutrient excretion (mg/d))/nutrient intake (mg/d) × 100. The determination of the apparent total instead of the ileal digestibility shows several limitations, especially with regard to microbial nutrient fermentation in the large intestine and endogenous nutrient secretion. However, for ethical reasons, nutritional studies in cats often consider the calculation of the apparent total instead of the ileal digestibility. In the present study, only faecal CP concentrations were measured, and it cannot be excluded that microbial fermentation of protein in the large intestine, especially in the case of a lower dietary protein quality, and endogenous amino acid secretion have falsified the results. Nevertheless, it is interesting to notice that the feed intake of the cats was higher when the low protein quality diets were fed, but the BW of the animals did not differ depending on the dietary protein quality. This observation indicates a lower energy utilisation in the low protein quality groups. On the other hand, the BW of the cats increased with increasing dietary protein concentrations, independently of the dietary protein quality. Although it could be speculated that the enhanced BW when feeding a high protein diet could be due to an underestimation of the energy intake by dietary protein, it should be considered that the differences in the BW of the cats were only small, and the data should be carefully interpreted. Overall, it seems that diets with a lower protein quality showed a greater feed acceptance, but a lower energy supply in the cats of the present study. As the study did not include measurements in respiratory chambers, the energy expenditure of the animals, however, could not be clearly determined. The present results can therefore provide some indications on the utilisation of dietary protein, but should be followed by comprehensive metabolic investigations.

With regard to the energy utilisation, it should not go unmentioned that varying dietary protein levels were necessarily accompanied by varying amounts of carbohydrates and fat in the experimental diets. Therefore, it cannot be excluded that the observed effects on the BW of the cats were partly attributed to changes in the intake of other macronutrients than protein. However, the energy density was comparable among the diets, ensuring a controlled protein intake of the cats. Moreover, a previous study could demonstrate that the energy balance in cats did not differ when feeding diets high in protein, fat or carbohydrates [30]. It can therefore be assumed that the observed effects of the diets on the BW of the cats were predominantly attributed to changes in the protein level and quality.

One limitation of the present study is that the effect of fibre on mineral bioavailability is not clear. In this respect, high dietary fibre concentrations can decrease the apparent digestibility of minerals [8]. Further, in our experience, the analysis of crude fibre content of diets can vary and, as a consequence, data should be interpreted with caution. In the present study, however, the apparent digestibility of most minerals was similar in all treatment groups, and the apparent digestibility of P was higher in the low protein quality groups with the slightly higher crude fibre concentrations. Thus, the observed differences in the apparent digestibility of P depending on the dietary protein quality require further investigations in future studies.

With regard to the evaluated effects of the dietary protein level and quality, it should finally be discussed that no baseline measurements were performed prior to feeding the experimental diets. This can be considered as a limitation of the study, as potential individual differences in the metabolism of the animals could not be included in the data analysis. However, all cats were clinically healthy prior and throughout the study, which is also underlined by the measured urea and creatinine concentrations in the blood of the cats. Thus, no impaired liver or kidney function of the cats can be assumed. In addition, the study design took potential animal-related metabolic differences into account by including a sufficiently high number of animals and feeding the experimental diets to each cat. Thus, individual variations in renal excretion or nutrient utilisation were reasonably considered by the present study, but could have been further improved by baseline measurements.

## Conclusions

In conclusion, feeding canned diets with varying protein concentrations to cats could not support observations in humans [32] and results of a previous study in cats with dry extruded diets [26] that a high protein intake increases the risk for CaOx urolith formation. In the present study, a high urine volume was observed by feeding the canned diets. The diluting effect possibly masked the described [26, 32] negative effects of a high protein diet on renal Ca, Ox and citrate excretion. In contrast, the high urine volume could not prevent high urinary RSS MAP values among all treatment groups. A lower dietary protein quality seemed to increase feed acceptance in cats, but to decrease energy utilisation.

## Additional file

Additional file 1: **Table S1.** Nutrient concentrations of the experimental diets, expressed on the basis of the dietary energy density. (DOCX 43 kb)

## Abbreviations

BW: Body weight
Ca: Calcium
CaOx: Calcium oxalate
CP: Crude protein
DM: Dry matter
K: Potassium
MAP: Magnesium ammonium phosphate
ME: Metabolisable energy
Mg: Magnesium
Na: Sodium
Ox: Oxalate
P: Phosphorus
RSS: Relative supersaturation

## References

[1] Colgrave ML, Allingham PG, Tyrrell K, Jones A (2012). Multiple reaction monitoring for the accurate quantification of amino acids: using hydroxyproline to estimate collagen content. Methods Mol Biol.

[2] Dijcker JC, Plantinga EA, van Baal J, Hendriks WH (2011). Influence of nutrition on feline calcium oxalate urolithiasis with emphasis on endogenous oxalate synthesis. Nutr Res Rev.

[3] Dijcker JC, Hagen-Plantinga EA, Hendriks WH (2012). Changes in dietary macronutrient profile do not appear to affect endogenous urinary oxalate excretion in healthy adult cats. Vet J.

[4] Dijcker JC, Hagen-Plantinga EA, Thomas DG, Queau Y, Biourge V, Hendriks WH (2014). The effect of dietary hydroxyproline and dietary oxalate on urinary oxalate excretion in cats. J Anim Sci.

[5] Funaba M, Yamate T, Hashida Y, Maki K, Gotoh K, Kaneko M (2003). Effects of a highprotein diet versus dietary supplementation with ammonium chloride on struvite crystal formation in urine of clinically normal cats. Am J Vet Res.

[6] Funaba M, Oka Y, Kobayashi S, Kaneko M, Yamamoto H, Namikawa K (2005). Evaluation of meat meal, chicken meal, and corn gluten meal as dietary sources of protein in dry cat food. Can J Vet Res.

[7] Gericke S, Kurmies B (1952). Colorimetrische Bestimmung der Phosphorsäure mit Vanadat-Molybdat (Colorimetric determination of phosphoric acid with vanadate molybdate). Fres Zeitsch Anal Chem.

[8] Gralak MA, Leontowicz M, Morawiec M, Bartnikowska E, Kulasek GW (1996). Comparison of the influence of dietary fibre sources with different proportions of soluble and insoluble fibre on Ca, Mg, Fe, Zn, Mn and Cu apparent absorption in rats. Arch Tierernahr.

[9] Hashimoto M, Funaba M, Abe M, Ohshima S (1995). Dietary protein levels affect water intake and urinary excretion of magnesium and phosphorus in laboratory cats. Exp Anim.

[10] Hashimoto M, Funaba M, Abe M, Ohshima S (1996). Effect of chronic high protein intake on magnesium, calcium, and phosphorus balance in growing cats. Exp Anim.

[11] Hawthorne AJ, Markwell PJ (2004). Dietary sodium promotes increased water intake and urine volume in cats. J Nutr.

[12] Hoffman JR, Falvo MJ (2004). Protein - Which is Best?. J Sports Sci Med.

[13] Houston DM, Elliott, DE. Nutritional management of feline lower urinary tract disorders. In: Pibot P, Biourge V, Elliott D, editors. Encyclopedia of feline clinical nutrition. Aimargues: Royal Canin; 2008. p. 285–321.

[14] Kirk CA, Ling GV, Franti CE, Scarlett JM (1995). Evaluation of factors associated with development of calcium oxalate urolithiasis in cats. J Am Vet Med Assoc.

[15] Lemann J, Pleuss JA, Gray RW, Hoffmann RG (1991). Potassium administration reduces and potassium deprivation increases urinary calcium excretion in healthy adults [corrected]. Kidney Int.

[16] Lemann J, Pleuss JA, Gray RW (1993). Potassium causes calcium retention in healthy adults. J Nutr.

[17] Liebman M, Chai W (1997). Effect of dietary calcium on urinary oxalate excretion after oxalate loads. Am J Clin Nutr.

[18] Liebman M, Costa G (2000). Effects of calcium and magnesium on urinary oxalate excretion after oxalate loads. J Urol.

[19] Lulich JP, Osborne CA, Carvalho M, Nakagawa Y (2012). Effects of a urolith prevention diet on urine compositions of glycosaminoglycans, Tamm-Horsfall glycoprotein, and nephrocalcin in cats with calcium oxalate urolithiasis. Am J Vet Res.

[20] Messia MC, Marconi E (2012). Innovative and rapid procedure for 4-hydroxyproline determination in meat-based foods. Methods Mol Biol.

[21] Morozumi M, Hossain RZ, Yamakawa K, Hokama S, Nishijima S, Oshiro Y, Uchida A, Sugaya K, Ogawa Y (2006). Gastrointestinal oxalic acid absorption in calcium-treated rats. Urol Res.

[22] National Research Council (NRC) (2006). Nutrient requirements of dogs and cats.

[23] Naumann C, Bassler C (2004). Die chemische Untersuchung von Futtermitteln 3. Aufl., 5. Ergänzungslieferung (Chemical Feed Analyses, Vol. 3).

[24] Osborne CA, Lulich JP, Thumchai R, Koehler L, Ulrich L (1995). Etiopathogenesis and therapy of feline calcium oxalate urolithiasis. Proceedings of the 13th Annual ACVIM Forum, Orlando, FL.

[25] Passlack N, Zentek J (2013). Urinary calcium and oxalate excretion in healthy adult cats are not affected by increasing dietary calcium levels. PLoS One.

[26] Paßlack N, Burmeier H, Brenten T, Neumann K, Zentek J (2014). Relevance of dietary protein concentration and quality as risk factors for the formation of calcium oxalate stones in cats. J Nutr Sci.

[27] Paßlack N, Brenten T, Neumann K, Zentek J (2014). Investigations on the effects of potassium chloride and potassium bicarbonate in the diet on the urinary pH and mineral excretion of adult cats. Br J Nutr.

[28] Paßlack N, Burmeier H, Brenten T, Neumann K, Zentek J (2014). Short term effects of increasing dietary salt concentrations on urine composition in healthy cats. Vet J.

[29] Penniston KL, Nakada SY (2009). Effect of dietary changes on urinary oxalate excretion and calcium oxalate supersaturation in patients with hyperoxaluric stone formation. Urology.

[30] Riond JL, Stiefel M, Wenk C, Wanner M (2003). Nutrition studies on protein and energy in domestic cats. J Anim Physiol Anim Nutr (Berl).

[31] Robertson WG, Jones JS, Heaton MA, Stevenson AE, Markwell PJ (2002). Predicting the crystallization potential of urine from cats and dogs with respect to calcium oxalate and magnesium ammonium phosphate (struvite). J Nutr.

[32] Robertson WG. Dietary recommendations and treatment of patients with recurrent idiopathic calcium stone disease. Urolithiasis. 2015; 10.1007/s00240-015-0849-2. 10.1007/s00240-015-0849-2 26645870

[33] Sabry ZI, Shadarevian SB, Cowan JW, Campbell JA (1965). Relationship of dietary intake of Sulphur amino-acids to urinary excretion of inorganic sulphate in man. Nature.

[34] Zentek J, Schulz A (2004). Urinary composition of cats is affected by the source of dietary protein. J Nutr.

