# Supplementary material for: Influence of protein concentration and quality in a canned diet on urine composition, apparent nutrient digestibility and energy supply in adult cats
Source: BMC Vet Res. 2018 Jul 25;14:225. doi: 10.1186/s12917-018-1517-x (PMC6060449; doi:10.1186/s12917-018-1517-x)
Supplement: Supplementary file 1 — Table S1. Nutrient concentrations of the experimental diets, expressed on the basis of the dietary energy density. (DOCX 43 kb) [file 12917_2018_1517_MOESM1_ESM.docx]

**Table S1.** Nutrient concentrations of the experimental diets, expressed on the basis of the dietary energy density.

| **Analysed composition** |  | **Low protein quality** | | | **High protein quality** | | |
| --- | --- | --- | --- | --- | --- | --- | --- |
|  |  | **36.7%** | **45.0%** | **56.1%** | **36.2%** | **43.3%** | **54.9%** |
| Crude protein | g/1000 kcal ME [g/MJ ME] | 75.5 [18.0] | 90.6 [21.6] | 115 [27.5] | 73.5 [17.5] | 85.0 [20.3] | 113 [27.0] |
| Crude fat | g/1000 kcal ME [g/MJ ME] | 58.3 [13.9] | 59.6 [14.2] | 55.6 [13.3] | 59.7 [14.3] | 62.5 [14.9] | 53.8 [12.9] |
| Crude fibre | g/1000 kcal ME [g/MJ ME] | 0.64 [0.15] | 1.42 [0.34] | 2.16 [0.52] | 1.37 [0.33] | 0.88 [0.21] | 1.39 [0.33] |
| Crude ash | g/1000 kcal ME [g/MJ ME] | 15.9 [3.79] | 14.9 [3.55] | 17.1 [4.10] | 13.9 [3.32] | 13.5 [3.23] | 15.5 [3.69] |
| Ca | g/1000 kcal ME [g/MJ ME] | 2.31 [0.55] | 2.30 [0.55] | 2.58 [0.62] | 2.17 [0.52] | 2.20 [0.53] | 2.42 [0.58] |
| P | g/1000 kcal ME [g/MJ ME] | 1.81 [0.43] | 1.68 [0.40] | 1.83 [0.44] | 1.57 [0.38] | 1.62 [0.39] | 1.79 [0.43] |
| Na | g/1000 kcal ME [g/MJ ME] | 1.20 [0.29] | 1.16 [0.28] | 1.69 [0.40] | 0.99 [0.24] | 1.14 [0.27] | 1.39 [0.33] |
| K | g/1000 kcal ME [g/MJ ME] | 2.27 [0.54] | 1.88 [0.45] | 2.56 [0.61] | 1.78 [0.42] | 1.69 [0.40] | 1.78 [0.43] |
| Mg | g/1000 kcal ME [g/MJ ME] | 0.10 [0.02] | 0.10 [0.02] | 0.10 [0.02] | 0.09 [0.02] | 0.09 [0.02] | 0.10 [0.02] |
| Ox | g/1000 kcal ME [g/MJ ME] | 0.05 [0.01] | 0.06 [0.01] | 0.07 [0.02] | 0.07 [0.02] | 0.07 [0.02] | 0.07 [0.02] |
| Nitrogen-free extracts | g/1000 kcal ME [g/MJ ME] | 55.3 [13.2] | 34.8 [8.32] | 15.4 [3.67] | 54.7 [13.1] | 34.6 [8.26] | 21.9 [5.23] |
